# Live demonstration versus multimedia: A comparison of two educational methods of orthodontic dental cast trimming 

**DOI:** 10.30476/JAMP.2021.91561.1450

**Published:** 2022-04

**Authors:** ALIREZA ALIZADEH, MOSTAFA SHEIKHI, MOHAMMAD MASOUD VAKILI, AZIN NOURIAN

**Affiliations:** 1 School of dentistry, Zanjan University of Medical Sciences, Zanjan, Iran; 2 Department of orthodontics, School of dentistry, Zanjan University of Medical Sciences, Zanjan, Iran; 3 Department of Health Education & Health Promotion, School of Public Health, Zanjan University of Medical Sciences, Zanjan, Iran

**Keywords:** Orthodontics, Multimedia, Education

## Abstract

**Introduction:**

The aim of this study was to compare the effect of two educational methods: live practical and multimedia demonstrations of dental cast trimming.

**Methods:**

In this quasi-experimental study, all 44 dental students from Zanjan University Dental School who were studying in the 8th semester entered the study. Using simple randomization, we assigned them to two groups. Multimedia and live demonstrations were used in the intervention (n=21) and control (n=23) groups, respectively. Knowledge of students was assessed using pre-test and post-test. Practical skills were compared using the students' final semester scores. The Individual Development and Educational Assessment questionnaire was used to assess the students' reaction to instruction. For data analysis, mean and standard deviation, and independent and paired t-tests were used. Statistical analysis was performed using SPSS software.

**Results:**

In terms of knowledge, the pre-test scores of the intervention and control groups were not significantly different (p-value= 0.457), and the post-test scores of the two intervention and control groups had no significant difference (p-value= 0.053); however, in both intervention and control groups there was a significant difference between the scores before and after the test, and the scores of both groups increased after training (p-value= 0.001, p-value= 0.001). In terms of practical skills, no significant difference was observed in the mean and standard deviation of the scores in the two groups (p-value=0.902). There was no significant difference in terms of the students' reaction to instruction.

**Conclusion:**

All students passed this course successfully. Further, their knowledge and skills were improved in both groups. Similar to the live practical demonstration, that of the multimedia led to a positive reaction to instruction in students. Therefore, multimedia education can be used well with the traditional method and even replaces it.

## Introduction

Orthodontic records are essential for diagnosis and treatment planning of orthodontic cases ( 1 ). Orthodontists have long been using patient's’ dental records to facilitate the assessment of dental position ( 2 ). Proper dental cast trimming has some advantages, including easier asymmetry diagnosis, and more acceptable presentation to the patients ( 3 ). 

Following the increasing growth of information and communication technology, traditional methods of education alone do not meet the educational needs of the new generation of students ( 4 ). One of the traditional teaching methods is lecturing, which is common in most universities in the country. In this method, the teacher explains the content orally ( 5 ). However, teaching is not automatically equivalent to learning. Learning is the acquisition of knowledge and developing skills to put that knowledge into practice and guide self-directed learning that must continue for a long time ( 6 ).

With development of the Internet, Internet-based education, known as e-learning, has attracted the attention of educators. E-learning is defined as using new Internet technologies to improve the quality of learning by facilitating access to resources and distance services using network technologies ( 7 ). One type of e-learning is multimedia education, which involves the creation of a mental imagery using words and images. The definition has a wide range that includes book-based environments containing texts and images, computer-based environments consisting of narration and animation, and virtual game environments consisting of interactive speech and animation ( 8 ).

Typically, dental education is based more on memorizing and recalling the teaching contents. The use of virtual learning tools is one of the new opportunities that advancement of information technology has provided for education. The advantages of this method include reduction of educational costs, possibility of teaching from anywhere and anytime, reproducibility of learning, and change from teacher-centered to student-centered education ( 9 ).

The sudden outbreak of Covid-19 posed many challenges to the world healthcare system; it also affected other areas, such as education ( 10 ). With the advent of the Covid-19 epidemic around the world, health protocols emphasized social distancing ( 11 ). Therefore, in many countries, face-to-face education in schools and universities was abandoned in order to reduce the spread of coronavirus, and e-learning became a necessity ( 12 , 13 ).

Atik et al. (2020) in a study entitled "The effect of live video training on wire bending in dental students" showed no significant difference in terms of skills between the two groups of live training and video training. There was also no difference in the level of student satisfaction between the two groups of students ( 14 ).

In 2019, Lima et al. conducted a systematic review study on the impact of the distance learning process on orthodontic education and concluded that distance education was effective and complimented the traditional education method ( 15 ). On the other hand, an increase in the level of awareness and satisfaction with the use of multimedia was also reported ( 16 , 17 ). The results of the study conducted by Gonipath et al. (2017) showed that video education, along with other teaching methods, had a better effect on the learning of dental students ( 18 ).

Few studies have compared the effect of multimedia and live demonstration on practical skills in dentistry, especially orthodontics and also considering the importance of using modern training methods ( 15 ).

Since few studies have compared the effect of multimedia and live demonstration on practical skills in dentistry, especially orthodontics and also considering the importance of using modern training methods, ( 15 ) This study aims:

1. To compare knowledge assessment test of live practical and multimedia training group before and after training,

2. To compare practical skill scores of dental cast trimming in the live practical and multimedia training groups,

3. To compare students' reaction to instruction in the live and multimedia practical training.

## Methods

**Figure 1:**
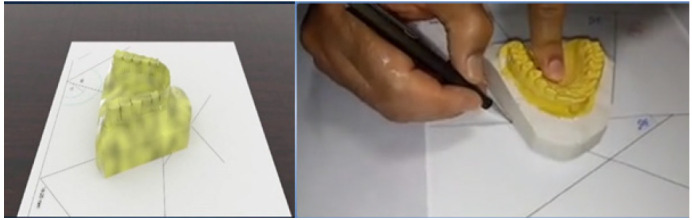
Multimedia was fabricated by Articulate Storyline version 3.5, and 3D animation was created by 3ds Max software 2018

The present quasi-experimental study was conducted in two parallel groups using pre-test and post-test. All 44 dental students from Zanjan University Dental School who were studying in the 8th semester and had chosen the first grade practical orthodontic course for the first time entered the study (total sampling). After obtaining informed consent to participate in the study, through simple random sampling, we assigned them to one of two groups: control (conventional live demonstration method) or intervention (multimedia training method). 23 students participated in the control group and 21 in the intervention group. This research was carried out during the years 2019 and 2020. Articulate Storyline version 3.5 was used to create multimedia features; also, 3D animation was created by 3ds Max software 2018 (Figure 1).

This study was approved by the ethics committee in Zanjan University of Medical Sciences with the code IR.ZUMS.REC.1398.390. The data collection tool for the pre-test and post-test was a simple researcher-made questionnaire consisting of 15 four-choice questions about dental cast trimming with one correct answer. The validity of the instrument was confirmed using the content validity method, which included the opinions of a five-member expert panel. Correct answers received a one-point score, while erroneous responses received a zero-point score. The reliability of the instrument was determined by calculating the Kuder-Richardson (0.85) in a sample of twenty 9th semester students who were similar to the target sample. 

Then, the training course was started, and students were trained in two groups of control and intervention. To avoid the possibility of the control group receiving the multimedia file, it was given to the students by department computers and could not be used at home. To standardize the conditions for all students, we selected a standard upper and lower dental cast approved by an orthodontist, and molding was prepared using ORMADUPLO (Dentari S.p.A) additive silicone according to the mold manufacturer's instructions. It was prepared from the casts and molded with Dental Model Stone TARA250 Type III (TARA). In total, 44 pairs of maxillary and mandibular dental casts were prepared equally for the students to trim. At the end of the training course, a 20-item standard objective scoring checklist was used to determine the effect of education on the students' practical skills. Scoring was done by an orthodontist blinded to the group allocation. The maximum score to be approved was 20, and the minimum was 12. Also, to determine the effect of education on the students' knowledge at the end of the course, we gave a four-choice post-test, with similar questions of the pre-test. At the end of the course, the students' reaction to instruction and course with the training course was evaluated using the standard Individual Development and Education Assessment (IDEA) questionnaire ( 19 ). The reliability and validity of the translated questionnaire were confirmed, citing the study of Nourian et al. ( 20 ). IDEA questionnaire had 5 domains with 47 questions on a Likert scale: 20 questions on the Instructor's teaching, 12 on ​​educational content, 3 associated with the ​​difficulty of the course, 7 on the ​​attitude towards the educational course, and 5 questions related to the final judgment of the students about the educational course. For data analysis, descriptive and analytical statistical methods including calculation of mean and standard deviation, as well as independent and paired t-tests were used, p-values ​​less than 0.05 were considered significant. Statistical analysis was performed using the Statistical Package for Social Sciences (SPSS) 17.0 (SPSS, Chicago, IL). 

## Results

Twenty four (54.5%) female and twenty one (45.5%) male students participated in this study. The mean age was 21.43±0.76 years (Table 1). According to the analysis of paired t-test, the mean of post-test scores in both intervention and control groups showed a significant increase compared to that of pre-test scores. The independent t-test showed that before and after training the scores of the two groups were not statistically significant (Table 2).

**Table 1:** demographic and baseline data in the two groups

Variables	Study (n= 21)	Control (n=23)	p
Age	21.38±0.59	21.47±0.89	0.443
Male/female	9/12	11/12	0.741

**Table 2:** The mean and standard deviation of knowledge scores in the study and control groups before and after the intervention

Knowledge score	Study (n= 21)	Control (n=23)	Mean difference (95%CI)	p
Before	6.95±2.10	6.56±1.23	0.38(-0.68 to 1.46)	0.457*
After	13.38±1.35	12.48±1.62	0.90(-0.01 to 1.81)	0.053
Mean difference (95%CI)	6.42(5.33 to 7.52)	5.91(5 to 6.82)	0.51(-0.85 to 1.88)	0.452
p	0.001**	0.001	-	-

*Based on independent sample t- test; **Based on paired t- test

All students in both groups received pass scores above 12. The mean and standard deviation scores of the students' practical skills in the control and intervention groups were 18.34±1.61 and 18.28±1.53, respectively; further, the scores of students in both groups were not significantly different (p-value= 0.902). 

The mean and standard deviation in both groups showed above-average positive students' reaction to the instruction and course; however, there was no significant difference between the two groups in terms of the instructor's teaching, educational content, course difficulty, attitude towards an educational course, and final judgment (Table 3). 

**Table 3:** Comparison of mean and standard deviation of students' reaction to instruction and course with dental cast trimming course in two intervention and control groups

Students' reaction to instruction and course domain	Study(n=21)	Control(n=23)	p
Instructor’s teaching	3.85±0.47	3.96±0.46	0.451
Educational content	3.97±0.46	3.83±0.52	0.357
Course difficulty	3.47±0.63	3.65±0.47	0.303
Attitude towards the educational course	3.96±0.51	3.89±0.40	0.641
Final judgement	4.02±0.30	3.70±0.71	0.061
Total	3.94±0.32	3.81±0.39	0.298

## Discussion

 In the present study, the level of students' knowledge assessed by knowledge test showed that the intervention group students who received multimedia training had higher scores than the group with live demonstration training by the teacher; however, the difference between the scores of the control and intervention groups was not significant statistically. In terms of the students' skills in dental cast trimming, the scores of the control group were slightly higher than those of the intervention group, but they were not statistically significant. Also, the two groups were not significantly different; both groups showed above-average positive reaction to the instruction and course. 

Atik et al. compared the wire bending process using live demonstration and instructional video; in their study, no significant difference was observed between the two groups in terms of satisfaction and skill test, which was consistent with the present study. However, in the present study, in addition to skill and reaction to instruction and course tests, a knowledge test was performed ( 14 ). Having reviewed 15 articles, Lima et al., concluded that there was no significant difference in terms of acceptability, satisfaction, and skills between the traditional education and e-learning groups, which is in line with the present study. However, this study did not examine the acceptability; thus, we do not comment on this issue (15). Al-Taweel et al. stated that satisfaction with technology-based education was moderate to low, which is contrary to the result of the present study ( 21 ). 

Subhash et al. used animation and PowerPoint teaching methods in comparison with the traditional method for the physiology course. They concluded that the score of students who received the first training was higher than those who received the traditional education in the long term and short term ( 16 ). The results of this research are not in line with those of the present study. Our course was practical while it was theoretical in Subhash's study. Moreover, in the present study, the control group received live demonstration, while in Subhash' study, they received lecture-based education. One of the strengths of the present research compared to Subhash' study was the comparative analysis of pre-test and post-test, as well as measuring the students' reaction to instruction and course.

Thilakmara et al. used video and live training to teach the laboratory process and concluded that the video training group scored better, which was not consistent with the result of the present study ( 22 ). The difference in results can be explained by the fact that in Thilkamara's study, students had access to video all the time with no place limit; however, in the live education group, the students were trained only by a professor at a specific time, affecting the scores of the students. In contrast, in the present study, in order to standardize the conditions, we allowed the students have access to multimedia education only at the department through university computers, so they had conditions almost equal to the control group; also, in addition to practical skills, the students' knowledge was assessed before and after training for more accurate evaluation. Mirkarimi et al. concluded that the practical scores of the traditional education and video-based groups were not statistically significant. They also assessed the students' satisfaction and found no significant difference between the two groups; their results were in line with those of the present study ( 23 ). However, the advantage of the present work over the mentioned study was the analysis of students' knowledge scores before and after education. According to Nourian et al., virtual education led to a positive attitude, and in general, the two groups were not significantly different in terms of reaction to instruction, except in ​​perception and judgment, with higher scores of the virtual education group. There was no significant difference between the virtual education and conventional groups in terms of knowledge ( 15 ); the results of this study were in line with those of the present research. 

One of the limitations of the present study is the small number of participants; thus, the authors suggest that more studies should be conducted in the future on the multimedia application in teaching other practical orthodontic skills on a larger sample size in different communities. 

## Conclusion

 At the end of the study, all students passed this course successfully. There was no statistically significant difference in the level of knowledge and practical skills of students about dental cast trimming using multimedia and live practical demonstration. Also, similar to the conventional method, multimedia-based teaching led to the students' positive reaction to instruction and course. Therefore, multimedia training can be a good alternative to that using the traditional approach; thus, it can be used in crises, such as during the Covid-19 pandemic. 

## Acknowledgement

The present article was extracted from a thesis approved and funded by the Faculty of Dentistry of Zanjan University of Medical Sciences (ethical code: IR.ZUMS.REC.1398.390). The authors would like to express their gratitude to the participants for their efforts and cooperation in this study.

**Conflict of Interest:** None Declared.
